# The readability of total elbow arthroplasty educational materials

**DOI:** 10.1016/j.jsea.2026.100071

**Published:** 2026-08-10

**Authors:** Ryan Terrany, William Cusma, Suleiman Sudah, Eric Kholodovsky

**Affiliations:** a Department of Orthopaedic Surgery, Robert Wood Johnson Medical School, New Brunswick, NJ, USA; b Department of Orthopaedic Surgery, Monmouth Medical Center, New Brunswick, NJ, USA; c Department of Orthopaedic Surgery, University of Miami Leonard M. Miller School of Medicine, Miami, FL, USA

**Keywords:** Total elbow arthroplasty, Patient education materials, Readability, Health literacy, Online health information, Clinical practice websites

## Abstract

**Background:**

The purpose of this study was to evaluate the readability of patient education materials related to total elbow arthroplasty (TEA) and to compare readability across clinical practice and general health information websites.

**Methods:**

A Google search using the term “Total Elbow Arthroplasty patient information” was performed in September 2025. The first 25 websites containing written patient educational materials on TEA were included, excluding websites with only graphics, videos, or tables. Websites were categorized as clinical practice or general health information sources. Non-educational content was removed, and the remaining text was then analyzed using 9 validated readability formulas to calculate grade level scores for each source. Comparisons between clinical practice and general health information websites were performed using independent sample *t*-tests.

**Results:**

Of the 25 patient education materials analyzed, 11 (44%) were from clinical practice websites and 14 (56%) from general health information websites. The mean reading level across all materials was 12.1 ± 2.5, ranging from 7.87 to 17.25. None of the educational materials met the recommended sixth-grade reading level set by the National Institutes of Health, American Medical Association, and the US Department of Health and Human Services. Clinical practice websites demonstrated significantly lower reading levels compared to general health information websites across most formulas (10.4 ± 2.2 vs. 13.4 ± 1.9; *P* = .002).

**Conclusion:**

Online patient educational materials for TEA are written well above recommended reading levels. Although clinical practice websites are comparatively more readable than general health information sites, both remain above recommended standards. These findings underscore the need for plain language revisions and patient-centered design to improve comprehension and support informed decision-making among TEA candidates.

Total elbow arthroplasty (TEA) is a surgical procedure used to treat a variety of elbow joint conditions including advanced rheumatoid arthritis, osteoarthritis, and complex fractures, aiming to alleviate pain, restore function, and improve mobility.^18^ TEA is a relatively uncommon procedure with an annual incidence of 1.4 per 100,000 people in Western countries.^11^ Despite its low utilization, TEA remains an important option for patients with advanced elbow pathology. Reported implant survival rates are 87.8% at 5 years and 70.7% at 10 years. However, TEA is associated with notable complication rates, including infection, aseptic loosening, instability, and periprosthetic fractures, with overall complication rates of 16.3% and revision rates of 14.6% at long-term follow-up.^10^ Compared to other joint arthroplasty procedures, TEA carries a higher risk of overall complications.^16^ Given these risks, it is essential that patients have a clear and comprehensive understanding of the procedure, its associated benefits, and potential complications.

Patients increasingly rely on the internet to self-educate on medical conditions and treatment options, both before and after discussion with a physician.^4^ General search engines, particularly Google, represent a primary information source for patients seeking medical information online. By entering symptoms, key diagnostic terms, and treatment protocols, users are directed to a wide range of sources, including online forums, advocacy groups, peer-reviewed journal articles, and hospital websites.^2^^,^^14^ Importantly, users tend to focus their reading primarily on the highest-ranked search results, making the readability and accessibility of these materials particularly critical.^14^ Patient education materials (PEMs) have emerged as a critical source of health information for individuals seeking to better understand their medical conditions. PEMs represent the second most frequently accessed source of medical information, surpassed only by direct consultation with a physician.^4^ PEMs typically provide information on the pathology overview, diagnosis, treatment options, and expected recovery related to a patient's condition, serving as a valuable adjunct to medical management.^3^^,^^4^^,^^14^ However, their effectiveness is contingent upon the patient's capacity to read, interpret, and comprehend the material presented.

PEMs authored by medical professionals often include technical language and specialized terminology that may limit comprehension among individuals without formal medical training. This phenomenon, referred to as "expert bias" in which clinicians overestimate patients' literacy and comprehension skills, has been documented across multiple medical disciplines.^1^^,^^3^^,^^18^ The accessibility of online health information does not necessarily translate to usability, as complex language can limit patients' ability to fully understand and apply the content to their own medical care.

The reading comprehension level of the population reading PEMs must be considered when they are created. The US Department of Health and Human Services reports that the average reading proficiency among American adults is at approximately an eighth- to ninth-grade level.^7^ Notably, approximately one-quarter of the US adults have low literacy and one-third have low numeracy, meaning that materials written for the “average” adult may be difficult for a large segment of the population to understand.^1^^,^^17^ To promote comprehension of information for the general population, the National Institutes of Health, American Medical Association, and the US Department of Health and Human Services recommend that PEMs be written in plain language, at a sixth-grade reading level or below.^1^^,^^17^^,^^19^ However, prior readability analyses of PEMs, both within orthopedic surgery and across other medical specialties, have consistently demonstrated that these recommendations are rarely met.^3^^,^^9^^,^^12^^,^^13^ The purpose of this study was to evaluate the readability of online PEMs related to TEA and to compare readability between clinical practice and general health information websites.

## Materials and methods

A Google search was conducted using the term "Total Elbow Arthroplasty patient information" in September of 2025. The search was performed using Google's advanced search settings, in which the options for "full sentence" and "English language" were selected. The search was conducted in a standard browser environment in incognito mode to minimize personalization of search results, and the first 25 websites that populated based on the search query were evaluated. Websites that included only graphics, videos, or tables and did not contain written educational content were excluded from this study.

Websites were classified into 2 categories: clinical practice and general health information websites as previously described.^9^ "Clinical practice" websites were defined as a health institution, imaging center, hospital, or private practice that provides medical or surgical treatment mainly to outpatients. "General health information website" was defined as a website from non-clinical institutions that provide general public health or medical information without directly offering patient care.^3^^,^^6^

The top 25 texts that met inclusion criteria regarding TEA were converted into a text-only format. Non-educational content, including webpage navigations, copyright notices, disclaimers, writer information, feedback questionnaires, links, website URLs, references, figures, tables, footnotes, addresses, and telephone numbers were removed to maintain consistency of scoring.

Readability scores were automatically calculated by transferring the texts to an online readability assessment tool (readabilityformulas.com). This software analyzes readability using the 9 validated tests: Average Reading Level Consensus Calculator, Flesch-Kincaid (FK) reading ease score, Gunning Fog Index, FK grade level, Coleman-Liau Index, Simple Measure of Gobbledygook (SMOG) Index, Automated Readability Index, original Linsear Write formula, and Linsear White grade level formula. A summary of the readability formulas is provided in Table I. Descriptive statistics were calculated for all readability metrics, including means and standard deviations. Comparisons between clinical practice and general health information websites were performed using independent sample *t*-tests. Statistical significance was set at *P* < .05. A Bonferroni correction was not applied given the exploratory nature of the comparisons across readability metrics; however, the consistency of findings across nearly all formulas supports the robustness of the observed differences. This study did not require institutional review board approval as it involved analysis of publicly available online materials and did not involve human subjects.

**Table I:** Readability tool calculation formulas.

Readability tool	Formula
Flesch-Kincaid reading ease score	Readability ease = 206.835−(1.015 × (total words ÷ total sentences)) - (84.6 × total syllables ÷ total words)
Gunning Fog	Grade level = 0.4 × [(words ÷ sentences) + (100 × (complex words ÷ words))]
Flesch-Kincaid grade level	Grade level = (0.39 × (total words ÷ total sentences)) + (11.8 × (total syllables ÷ total words)) − 15.59
The Coleman-Liau Readability Index	Grade level = (0.0588 ∗ (total letters/total words) ∗ 100) - (0.296 ∗ (total sentences/total words) ∗ 100) - 15.8
SMOG Index	Grade level = 1.043×polysyllables×sentences30+3.1291
Automated Readability Index	Grade level = 4.71 × (characters/words) + 0.5 × (words/sentences)−21.43
Linsear Write grade level formula	Initial score = (total easy words + (3 ∗ total hard words))/total sentences Where: hard words are considered words with more than 2 syllables. Adjust the score: If the initial score is greater than 20, divide it by 2. Example: If the initial score is 21, then the adjusted score is 10.5. If the initial score is 20 or less, subtract 2 and then divide by 2. Example: If the initial score is 18, then the adjusted score is (initial score - 2)/2 = 8. Final score matches a the US grade level
Original Linsear Write formula	Reading score = ((total one-syllable words - total ignored words) + (3 ∗ (total standard sentences + total compound sentences))/(total words/100))

*SMOG*, Simple Measure of Gobbledygook.

## Results

After applying the inclusion and exclusion criteria, the first 25 websites were investigated, of which 11 were PEMs articles from clinical practice websites and 14 were PEMs from general health information websites. The titles and hyperlinks for the 25 included websites are listed in Table II, arranged in the order that they appeared on the Google search.

**Table II:** Titles and hyperlinks for the 25 included general health information and clinical practice websites.

Source number	PEM	Source type
1	https://www.orthobullets.com/shoulder-and-elbow/3089/total-elbow-arthroplasty	General health info
2	https://orthoinfo.aaos.org/en/treatment/total-elbow-replacement/	General health info
3	https://pmc.ncbi.nlm.nih.gov/articles/PMC6867907/	General health info
4	https://my.clevelandclinic.org/health/procedures/elbow-replacement-surgery	Clinical practice
5	https://www.zimmerbiomet.com/en/products-and-solutions/specialties/elbow.html	General health info
6	https://surgeryreference.aofoundation.org/orthopedic-trauma/adult-trauma/distal-humerus/basic-technique/total-elbow-arthroplasty	General health info
7	https://openorthopaedicsjournal.com/VOLUME/5/PAGE/115/FULLTEXT/	General health info
8	https://www.mayoclinic.org/tests-procedures/elbow-replacement-surgery/about/pac-20385126	Clinical practice
9	https://pmc.ncbi.nlm.nih.gov/articles/PMC4716570/	General health info
10	https://www.brighamandwomens.org/assets/bwh/patients-and-families/rehabilitation-services/pdfs/elbow-total-elbow-arthroplasty-bwh.pdf	Clinical practice
11	https://www.orthobullets.com/shoulder-and-elbow/422838/revision-total-elbow-arthroplasty	General health info
12	https://www.mountsinai.org/health-library/surgery/elbow-replacement	Clinical practice
13	https://www.mayoclinic.org/medical-professionals/orthopedic-surgery/news/challenging-elbow-revisions-total-elbow-arthroplasty-with-massive-bone-loss/mac-20541845	Clinical practice
14	https://www.sciencedirect.com/science/article/pii/S1877056815001747	General health info
15	https://www.physio-pedia.com/Total_Elbow_Arthroplasty	General health info
16	https://journals.lww.com/jaaosglobal/fulltext/2024/07000/influence_of_training_background_on_elbow.3.aspx	General health info
17	https://radiopaedia.org/articles/elbow-arthroplasty?lang=us	General health info
18	https://www.donalddolcemd.com/total-elbow-replacement-orthopedic-surgeon-fort-worth-texas.html	Clinical practice
19	https://www.sciencedirect.com/science/article/abs/pii/S0363502309001634	General health info
20	https://orthowisconsin.com/specialties/joint-replacement/total-elbow-replacement/	Clinical practice
21	https://hughston.com/wellness/total-elbow-arthroplasty-questions-for-a-specialist/	Clinical practice
22	https://www.methodisthealthsystem.org/methodist-orthopaedic-surgical-associates-mg/our-procedures/elbow/total-elbow-replacement/	Clinical practice
23	https://www.oint.org/total-elbow-replacement-frisco-mckinney-dallas-tx.html	Clinical practice
24	https://www.arrchorthopaedics.com/elbowarthroplasty.html	General health info
25	https://www.rnoh.nhs.uk/patients-and-visitors/patient-information-guides/total-elbow-replacement-patients-guide	Clinical practice

*PEM*, patient education material; *info*, information.

Readability analysis revealed a mean reading level of 12.1 ± 2.5 across all 9 validated readability formulas. Individual readability scores for each website are summarized in Table III. The range of scores varied across websites, with the lowest average reading level observed at 7.87 and the highest at 17.25, demonstrating considerable variability in complexity among PEMs regarding TEA. Importantly, none of the 25 PEMs (0%) met the sixth-grade reading level recommended by the National Institutes of Health, American Medical Association, and the US Department of Health and Human Services.^1^^,^^17^^,^^19^

**Table III:** Individual and mean scores of the top 25 PEMs on TEA

Website number	Source type	Average Reading Level Consensus Calculator	Automated Readability Index	Flesch reading ease score	Gunning Fog	Flesch-Kincaid grade level	Coleman-Liau Index	SMOG Index	Original Linsear Write formula	Linsear Write grade level formula
1	General health info	11.32	10.95	28	14.2	10.84	15.04	7.73	90	26.77
2	General health info	11.29	11.42	50	12.2	10.65	12.01	10.24	69	10.85
3	General health info	15.34	18.52	30	16.1	15.41	15.59	14.24	53	16.85
4	Clinical practice	8.71	8.12	61	10.3	7.5	10.82	7.73	83	10.22
5	General health info	13.64	12.99	27	15.3	12.82	15.52	11.34	58	18.57
6	General health info	11.42	10.02	44	12.6	10.17	12.84	9.29	65	11.46
7	General health info	15.42	17.14	26	16.8	15.39	16.01	14.09	51	15.12
8	Clinical practice	7.98	6.64	64	9.4	6.73	9.36	7.24	83	6.81
9	General health info	13.55	14.93	37	14	13.12	14.3	12.01	59	12.86
10	Clinical practice	13.37	13.96	32	14.6	12.67	15.41	11.43	60	11.17
11	General health info	11.2	10.64	32	13.9	10.44	14.85	8.05	85	29.4
12	Clinical practice	7.87	6.62	64	9.5	6.74	9.51	7.21	86	8.83
13	Clinical practice	11.89	12.12	49	12.5	10.44	13.02	10.18	68	10.39
14	General health info	13.47	14.69	43	14.2	12.58	12.91	12.39	51	12.3
15	General health info	14.05	13.82	20	16.2	13.64	16.78	11.42	63	17.82
16	General health info	17.75	22.92	15	17.6	19.02	17.8	16.4	44	21.6
17	General health info	13.08	12.43	27	16	11.7	16.54	9.9	72	18.18
18	Clinical practice	10.19	8.99	55	11	8.83	11.27	8.72	69	11.02
19	General health info	13.22	12.6	30	14.7	12.52	15.22	11.01	57	13.03
20	Clinical practice	12.39	12.6	36	12.3	11.45	15.61	9.75	66	14.62
21	Clinical practice	12.65	13.14	43	13.5	12.14	13.02	11.27	61	11.78
22	Clinical practice	8.51	7.36	63	10.5	7.51	9.5	8.23	84	10.69
23	Clinical practice	12.59	12.61	44	13.2	11.64	13.22	10.97	58	11.5
24	General health info	13.24	12.7	28	14.8	12.17	16.81	10.21	65	18.98
25	Clinical practice	8.64	7.94	62	10.1	7.77	10.05	8.13	81	11.24
Mean		12.1112	12.2348	40.4	13.42	11.3556	13.7204	10.3672	67.24	14.4824
Standard deviation		2.473147994	3.768536542	14.80146389	2.342541355	2.91146166	2.545694863	2.327075275	12.77458414	5.452744172
Min		7.87	6.62	15	9.4	6.73	9.36	7.21	44	6.81
Max		17.75	22.92	64	17.6	19.02	17.8	16.4	90	29.4

*TEA*, total elbow arthroplasty; *PEM*, patient education material; *SMOG*, Simple Measure of Gobbledygook; *min*, minimum; *max*, maximum; *info*, information.

When comparing clinical practice versus general health information websites, clinical practice PEMs had a significantly lower reading level across most readability metrics (Table IV). Clinical practice websites demonstrated lower Average Reading Level Consensus (10.4 ± 2.2 vs. 13.4 ± 1.9; *P* = .002), Automated Readability Index (10.0 ± 2.9 vs. 14.0 ± 3.5; *P* = .005), Gunning Fog Index (11.5 ± 1.8 vs. 14.9 ± 1.5; *P* < .001), FK grade level (9.4 ± 2.3 vs. 12.9 ± 2.4; *P* = .001), Coleman-Liau Index (11.9 ± 2.3 vs. 15.2 ± 1.7; *P* < .001), SMOG Index (9.2 ± 1.6 vs. 11.3 ± 2.4; *P* = .020), and Linsear Write grade level formula (10.8 ± 1.9 vs. 17.4 ± 5.6; *P* < .001). The Flesch reading ease score, where higher values indicate easier readability, was significantly higher for clinical practice websites (52.1 ± 11.9 vs. 31.2 ± 9.5; *P* < .001). Only the original Linsear Write formula did not demonstrate a statistically significant difference between website types (72.6 ± 10.9 vs. 63.0 ± 12.9; *P* = .07).

**Table IV:** Comparison between general health info and clinical practice reading levels.

Metric	General health info mean	General health info STD	Clinical practice mean	Clinical practice STD	*P* value
Average Reading Level Consensus Calculator	13.4	1.9	10.4	2.2	.002∗
Automated Readability Index	14	3.5	10	2.9	.005∗
Flesch reading ease score	31.2	9.5	52.1	11.9	<.001∗
Gunning Fog	14.9	1.5	11.5	1.8	<.001∗
Flesch-Kincaid grade level	12.9	2.4	9.4	2.3	.001∗
Coleman-Liau Index	15.2	1.7	11.9	2.3	<.001∗
SMOG Index	11.3	2.4	9.2	1.6	.020∗
Original Linsear Write formula	63	12.9	72.6	10.9	.07
Linsear Write Grade Level formula	17.4	5.6	10.8	1.9	<.001∗

*SMOG*, Simple Measure of Gobbledygook; *info*, information; *STD*, standard deviation.

Statistically significant values are denoted with an asterisk.

## Discussion

This study evaluated the readability of online PEMs related to TEA. The data demonstrate that online PEMs related to TEA are written at reading levels substantially above the sixth-grade reading level recommended by the National Institutes of Health, American Medical Association, and the US Department of Health and Human Services for patient comprehension.^1^^,^^17^^,^^19^ Among the 25 PEMs analyzed, the mean reading grade across all 9 validated readability metrics was 12.1 ± 2.5 (Table III). Importantly, none of the 25 PEMs analyzed (0%) met the recommended sixth-grade reading level threshold, with the lowest reading level observed at 7.87 and the highest at 17.25 (Table III), highlighting considerable variability in complexity across available materials.

When comparing clinical practice and general health information websites, clinical practice PEMs consistently demonstrated lower, and therefore more accessible, reading levels across most metrics (Table IV). For instance, the Average Reading Level Consensus was significantly lower for clinical practice sites compared with general health information sites, and the Automated Readability Index was similarly lower. Clinical practice websites also had higher Flesch reading ease scores, indicating easier readability. Additional measures, including the Gunning Fog Index, FK grade level, Coleman-Liau Index, SMOG Index, and Linsear Write grade level formula further supported this finding, with all demonstrating significantly lower reading levels for clinical practice sources (Table IV).

Interpretation of these findings in the context of prior literature is complicated by differences in how website categories are defined. In the present study, “clinical practice” websites included a broad range of patient care entities, including hospitals, outpatient practices, and diagnostic centers. In contrast, previous work has analyzed hospitals and clinical practices as distinct categories, reporting that hospital-based materials demonstrated lower readability levels than those from clinical practices and professional societies.^6^ These differences may reflect the influence of institutional resources, such as multidisciplinary collaboration and library support, which are more commonly available in hospital settings. As a result, the inclusion of hospitals within the “clinical practice” category in this analysis may obscure meaningful variation within this group. Taken together, these findings suggest that readability is shaped by multiple factors, including both institutional infrastructure and direct clinician–patient interaction, and that categorization strategies may significantly influence observed trends.

In addition to variability within clinical practice categories, heterogeneity within general health information sources must also be considered. In the present study, general health information sources included both consumer-oriented health websites and peer-reviewed research articles. The inclusion of peer-reviewed research articles, which are not typically intended for patient audiences and frequently employ technical language and domain-specific terminology, may have contributed to elevated readability scores within this group. Several of the websites classified as general health information sources, such as PubMed, ScienceDirect, and journal websites, represent peer-reviewed literature written for professional readers rather than patient-facing education content. Their appearance in search results reflects the reality that patients may encounter these materials when searching for health information; however, their inclusion may inflate the mean readability scores for the general health information category and widen the observed difference between groups. As a result, the general health information category may reflect a broader spectrum of communication styles compared to clinical practice websites, which are more consistently designed for patient interaction. This distinction highlights the importance of considering intended audience and content type when evaluating readability across online health information sources.

Notably, both clinical practice and general health information materials substantially exceeded recommended readability standards, underscoring the widespread nature of this issue. These results are consistent with prior literature in orthopedics and other medical specialties, which has shown that high reading complexity is common throughout online medical and surgical educational content. For example, studies evaluating PEMs for orthopedic conditions from the American Academy of Orthopaedic Surgeons and academic medical centers found mean reading levels between 9.2 and 14.8 grade levels, with the vast majority (81-97%) exceeding the recommended sixth-grade reading level.^9^^,^^12^^,^^13^ Approximately one-quarter of the US adults have low literacy, and the average reading level is eighth to ninth grade, suggesting that materials written above this level may be difficult to understand for a substantial portion of their intended readers.^17^ This discrepancy has important clinical implications, as TEA is a complex procedure associated with relatively high complication and revision rates, and inadequate patient comprehension may influence expectation setting, shared decision-making and patient recovery. Limited health literacy has been associated with worse pre-operative function, lower surgical expectations, decreased satisfaction, and prolonged hospitalizations in total joint arthroplasty populations. Across broader surgical populations, low health literacy is independently associated with increased post-operative complications, longer hospital stays, higher readmission rates, poorer adherence to treatment plans, and reduced adherence to enhanced recovery protocols.^7^^,^^15^^,^^18^

Based on these findings, the following recommendations are proposed for health-care institutions and medical societies: (1) establish readability standards for all patient-facing materials with target reading levels at or below sixth grade, (2) incorporate health literacy experts and patient representatives in the development and review of educational materials, (3) utilize automated readability assessment tools during the content creation process, and (4) conduct periodic audits of existing materials to ensure maintenance of readability standards.

Several factors likely contribute to the elevated reading levels observed in TEA PEMs. Orthopedic content inherently involves specialized terminology and procedural descriptions that are challenging to simplify without compromising accuracy. Additionally, materials authored by clinicians may reflect "expert bias," in which authors overestimate patients' literacy and comprehension skills, a phenomenon documented across multiple medical disciplines. The Agency for Healthcare Research and Quality and multiple professional organizations recommend systematic application of plain language principles to improve readability, including using common words instead of medical jargon, keeping sentences short (under 15 words), defining necessary medical terms, and incorporating visual aids.^1^^,^^19^ Evidence indicates that targeted simplification of polysyllabic words and complex syntax, combined with visual aids and summaries, can meaningfully lower reading levels and improve comprehension. Hill-Briggs et al^8^ demonstrated that systematic targeting of sentence length, active voice, and syllable reduction can decrease reading levels from 10 to 11th grade to below fifth grade while maintaining content accuracy. Practical strategies include replacing medical jargon with plain language alternatives when appropriate, defining unavoidable technical terms, using shorter sentences, organizing information with headings or bullet points, and incorporating illustrations to reinforce key concepts. While simplified materials have been shown to improve patient comprehension and preference, evidence regarding their impact on long-term behavioral adherence and clinical outcomes remains limited and requires further investigation.

This study has limitations that should be acknowledged. Although the readability metrics used in this study demonstrated a high degree of concordance, they were not entirely consistent. At present, there is no consensus regarding a single superior readability metric, which may contribute to variability in readability assessment. Different aspects of the written text influence the collected composite scores and metrics. For instance, FK grade levels are more heavily influenced by sentence length and syllable count, whereas the Automated Readability Index is more influenced by the ratio of characters to words. By employing 9 readability assessments, this study achieved a more thorough and balanced evaluation of readability.

Additionally, readability alone does not fully capture the overall quality or effectiveness of patient educational materials. Readability remains an important factor influencing patients' ability to comprehend medical information.^1^ Readability formulas evaluate only written text and do not account for patient comprehension, health literacy, document organization, or the contribution of visual elements such as figures, diagrams, tables, and formatting, all of which may substantially influence understanding.^1^ This limitation is particularly relevant in orthopedic surgery, where illustrations and anatomical diagrams are frequently used to explain complex anatomy, surgical procedures, implants, and post-operative expectations. Consequently, the exclusion of visual content from this analysis may underestimate the overall educational value of some PEMs. These findings should therefore be interpreted as an assessment of textual readability rather than the overall effectiveness of these resources in facilitating patient understanding. Future studies should incorporate validated measures of understandability and actionability, such as the Patient Education Materials Assessment Tool, to better evaluate the combined impact of written and visual educational content. Furthermore, digital health interventions and multimedia resources show promise as adjuncts to traditional written materials and may further enhance health literacy among orthopedic arthroplasty patients.^5^ This study focused exclusively on English language materials from a single search engine and did not evaluate whether patients actually access or utilize these online materials in their decision-making processes. The findings may not be generalizable to non-English speaking populations or to patients who access information through alternative search engines or social media platforms. Additionally, the sample size of 25 websites, while consistent with prior readability studies in orthopedic surgery, limits the statistical power of sub-group comparisons and may not fully represent the breadth of available online content. Finally, the search was conducted at a single time point in September 2025, and website contents may change over time.

Although improving readability is an important step toward enhancing patient education, readability alone does not guarantee comprehension, particularly for complex procedures such as TEA. Surgical decision-making requires patients to understand nuanced concepts including implant longevity, complication risks, post-operative restrictions, and revision surgery, which may not be fully conveyed through written educational materials regardless of reading level. Consequently, PEMs should be viewed as an adjunct to comprehensive discussions between patients and their treating surgeons rather than a replacement for these discussions. Future studies should evaluate not only readability but also patient comprehension, knowledge retention, and decision quality following exposure to revised educational materials.

## Conclusion

Patient educational materials on TEA consistently exceed the recommended sixth-grade reading level. Given the complexity of the procedure and its relatively high rates of complications and revisions, surgeons should recognize the potential gap between what patients read online and what they comprehend and proactively engage in patient-centered discussions to ensure shared decision-making in the clinical setting.

## Disclaimers:

Funding: No funding was disclosed by the authors.

Conflicts of interest: The author, their immediate family, and any research foundation with which they are affiliated have not received any financial payments or other benefits from any commercial entity related to the subject of this article.
